# Solubility, Permeability, Anti-Inflammatory Action and In Vivo Pharmacokinetic Properties of Several Mechanochemically Obtained Pharmaceutical Solid Dispersions of Nimesulide

**DOI:** 10.3390/molecules26061513

**Published:** 2021-03-10

**Authors:** Wei Wei, Veronica I. Evseenko, Mikhail V. Khvostov, Sergey A. Borisov, Tatyana G. Tolstikova, Nikolay E. Polyakov, Aleksandr V. Dushkin, Wenhao Xu, Lu Min, Weike Su

**Affiliations:** 1 National Engineering Research Center for Process Development of Active Pharmaceutical Ingredients, Collaborative Innovation Center of Yangtze River Delta Region Green Pharmaceuticals, Zhejiang University of Technology, Hangzhou 310014, China; 13619587845@163.com (W.W.); avd@ngs.ru (A.V.D.); xuwenhao@zjut.edu.cn (W.X.); yaolilumin@163.com (L.M.); 2 Institute of Solid State Chemistry and Mechanochemistry, Kutateladze, 18, Novosibirsk 630128, Russia; evseenko@solid.nsc.ru (V.I.E.); khvostov@nioch.nsc.ru (M.V.K.); polyakov@kinetics.nsc.ru (N.E.P.); 3 N. N. Vorozhtsov Novosibirsk Institute of Organic Chemistry, Lavrentiev Avenue 9, Novosibirsk 630090, Russia; sergalborisov@mail.ru (S.A.B.); tolstiktg@nioch.nsc.ru (T.G.T.); 4 Voevodsky Institute of Chemical Kinetics and Combustion, Institutskaya Str. 3, Novosibirsk 630090, Russia

**Keywords:** nimesulide, solid pharmaceutical dispersions, supramolecular complexes, micelles, disodium salt of glycyrrhizic acid, polysaccharide arabinogalactan, hydroxypropyl-β-cyclodextrin, magnesium carbonate, complexation, intrinsic solubility, bioavailability, anti-inflammatory action, pharmacokinetics

## Abstract

Nimesulide (NIM, *N*-(4-nitro-2-phenoxyphenyl)methanesulfonamide) is a relatively new nonsteroidal anti-inflammatory analgesic drug. It is practically insoluble in water (<0.02 mg/mL). This very poor aqueous solubility of the drug may lead to low bioavailability. The objective of the present study was to investigate the possibility of improving the solubility and the bioavailability of NIM via complexation with polysaccharide arabinogalactan (AG), disodium salt of glycyrrhizic acid (Na_2_GA), hydroxypropyl-β-cyclodextrin (HP-β-CD) and MgCO_3_. Solid dispersions (SD) have been prepared using a mechanochemical technique. The physical properties of nimesulide SD in solid state were characterized by differential scanning calorimetry and X-ray diffraction studies. The characteristics of the water solutions which form from the obtained solid dispersions were analyzed by reverse phase and gel permeation HPLC. It was shown that solubility increases for all complexes under investigation. These phenomena are obliged by complexation with auxiliary substances, which was shown by ^1^H-NMR relaxation methods. The parallel artificial membrane permeability assay (PAMPA) was used for predicting passive intestinal absorption. Results showed that mechanochemically obtained complexes with polysaccharide AG, Na_2_GA, and HP-β-CD enhanced permeation of NIM across an artificial membrane compared to that of the pure NIM. The complexes were examined for anti-inflammatory activity on a model of histamine edema. The substances were administered *per os* to CD-1 mice. As a result, it was found that all investigated complexes dose-dependently reduce the degree of inflammation. The best results were obtained for the complexes of NIM with Na_2_GA and HP-β-CD. In noted case the inflammation can be diminished up to 2-fold at equal doses of NIM.

## 1. Introduction

**Figure 1:**
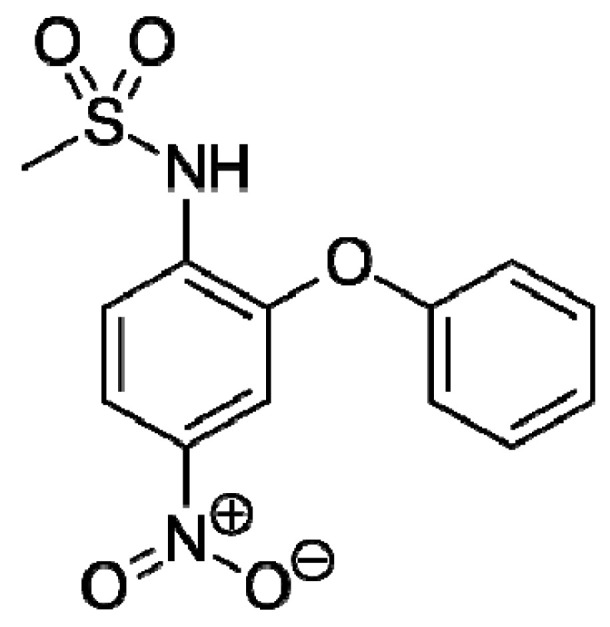
Structure of the nimesulide molecule.

Nimesulide (NIM, Figure 1) is a non-steroidal anti-inflammatory drug (NSAID) from the sulfonamides class. Its approved indications are treatment of acute pain, symptomatic treatment of osteoarthritis, and primary dysmenorrhea in adults and adolescents over 12 years of age.

NIM is a selective competitive inhibitor of cyclooxygenase-2 (COX-2), an enzyme involved in the synthesis of prostaglandins, mediators of edema, inflammation, and pain. It has anti-inflammatory, analgesic and antipyretic effects. It reversibly inhibits the formation of prostaglandin E2, both in the focus of inflammation and in the ascending pathways of the nociceptive system, including the pathways of spinal cord pain impulses. It also reduces the concentration of short-lived prostaglandin H2, from which prostaglandin E2 is formed under the action of prostaglandin isomerase. A decrease in the concentration of prostaglandin E2 leads to a decrease in the degree of activation of prostanoid EP-type receptors, which is expressed in analgesic and anti-inflammatory effects.

In terms of gastrointestinal safety, NIM is comparable to other NSAIDs. Due to concerns about the risk of hepatotoxicity, NIM has been banned in several countries (Spain, Finland, Belgium, Ireland, and the United States). Liver problems can lead to death and the need for liver transplants. This can happen as little as three days after starting treatment. The European pharmacovigilance database shows that NIM causes more cases of severe liver damage compared to other NSAIDs, as well as more cases of liver damage compared to COX-2 inhibitors [1,2,3].

NIM belongs to the II class of drugs, according to the biopharmaceutical classification system [4,5,6] and is characterized by low water solubility (~0.02 g/L), which slows its release from solid oral dosage forms, thus slowing the absorption of its dosage from the gastrointestinal tract into the bloodstream. This creates prerequisites for the use in medical practice of excessive dosages of nimesulide and the occurrence of the above undesirable side effects.

In our opinion, the improvement of NIM’s bioavailability by increasing its water solubility and accelerating absorption will promote the dose reduction along with high pharmacological effect preservation [7]. In our previous studies [8,9,10,11,12] we had shown that the usage of such supramolecular delivery systems is a successful to reduce the doses and toxicity of several other drugs. Additionally, we should note, that some auxiliary substances used, namely polysaccharide AG and glycyrrhizic acid (as far as its salts), possess hepatoprotective activity itself [13,14].

There are many articles devoted to increasing of NIM’s solubility, for example by using β-cyclodextrin, hydroxypropyl-β-cyclodextrin (HP-β-CD), [15,16,17] dendrimers, phosphatidylcholine [18,19] and solid dispersions with PVP, PEG [20,21]. The preparation of NIM complexes with metals Zn(II), Cu(II), Fe(III) and Sb(III) were also described [22]. In most cases only solubility and dissolution aspects and some in vitro experiments were published. An increase in the anti-inflammatory activity of NIM by complexation with methyl-β-cyclodextrin has been shown in [23]. Unfortunately, methyl-β-cyclodextrin has very limited use in pharmacy due to its high cost and not always desirable ability to extract cholesterol from cell membranes. The above researchers used various techniques for obtaining complexes and solid dispersions—dry and wet ball milling, kneading and liquid-phase synthesis.

The purpose of our investigations was to obtain solid pharmaceutical dispersions (SD) of NIM with HP-β-CD, plant polysaccharide arabinogalactan (AG), disodium salt of glycyrrhizic acid (Na_2_GA) and magnesium carbonate (MgCO_3_) in order to improve its solubility, membrane permeability, bioavailability and anti-inflammatory action (in vivo). We used mechanochemical treatment—dry milling for preparation of noted materials, which has advantages compare to traditional synthesis, which requires participation of liquid phase. Solid dispersion of NIM with noted excipients represents the most exciting research and development field related to amorphous pharmaceutical products. Two major distinct processes are usually widely used to prepare amorphous materials: solvent evaporation and melting. However, in the case of solvent evaporation method, organic solvents may be difficult to be removed from the final product, which can be especially concerning when highly toxic solvents are required to be employed. The major drawback of melting processes is high temperatures that may induce drug degradation or decomposition. In addition, melting processes require drug solubility/miscibility, which can be very difficult to achieve for some hydrophobic molecules. To the best of our knowledge, there are few studies on SD of NIM prepared by mechanical ball milling process. Recently, mechanochemistry has become an important subject of interest in pharmaceutical sciences for its role in the development of green synthesis [24,25,26,27], supramolecular structures [8], cocrystal synthesis [28], and amorphous SDs [9]. When a solid molecular compound is subjected to high energy mill, its structural and microstructural characters change considerably as well as its physical and chemical stability. All these changes may have a significant impact on biopharmaceutical properties, e.g., enhanced solubility and bioavailability [29]. No solvent or high temperature is needed during the process of mechanochemical treatment.

HP-β-CD is well known cyclic oligosaccharide, used in pharmacy for solubilization of lipophilic drug molecules [30]. Relative to other β-cyclodextrins it has advantages of higher water solubility and less toxic effects for oral application. Its complexes with NIM were described earlier [15,16,17]. Unfortunately, only the physico-chemical and solubility and dissolution aspects were investigated and no biology action was described. We used HP-β-CD for complexation with NIM as well-known reference for comparison with other excipients discussed in our article.

AG is a good water-soluble polysaccharide isolated from the wood of larch *Larix sibirica* and *Larix gmelinii*, which is a natural polysaccharide polymer composed of galactose and arabinose fragments consisting of a highly substituted backbone of 1–3 linked β-d-galactopyranose units with side chains of galactose and arabinose with total molecular weight 14–20 kDa [31,32]. In this case, the branched structure of AG macromolecules is especially favorable for complexing drug molecules [8,33,34,35]. As a result of such complexation the following positive changes in drugs’ toxic-pharmacological properties were obtained: increase of water solubility, effective dose and toxic side effects reduction [8,9,10,11,36].

Glycyrrhizic acid (GA) is a triterpene glycoside extracted from licorice root that demonstrates antiviral, anti-inflammatory, and anticancer properties [37,38]. Due to its amphiphilic properties, GA is capable of forming complexes and micelles [39] with a variety of hydrophobic molecules, substantially increasing their solubility, enhancing the permeability of drugs through cell membranes and their bioavailability [12,40,41,42,43,44,45,46]. In addition, disodium glycyrrhizin (Na_2_GA) is a salt of GA which can undergo hydrolysis in aqueous solutions and form free GA. Its advantage is that the solution formed has lower viscosity in contrast with GA solutions as far as its potassium salts are concerned. We can expect a synergetic effect of using Na_2_GA as a drug delivery system for NIM. Given that mechanochemical technology and Na_2_GA have the potential to improve the bioavailability of water insoluble drugs, we then evaluate bioavailability and bio-efficacy of NIM as an amorphous SD in a matrix consisting of Na_2_GA.

We have also used another approach for solubilization of NIM taking advantage of its acid nature and possible salt formation [7]. It known that acidity of NIM active pharmaceutical substance (API) is pK_a_ = 6.56 ± 0.01 [47]. In this way we obtained a solid dispersion with an alkali–pharmacopeia grade magnesium carbonate (MgCO_3)_. We assumed that release of NIM into water solution should be in ionized form as it is more soluble then neutral molecules.

## 2. Results and Discussion

### 2.1. Physical Characterization Studies of NIM SD

**Figure 2:**
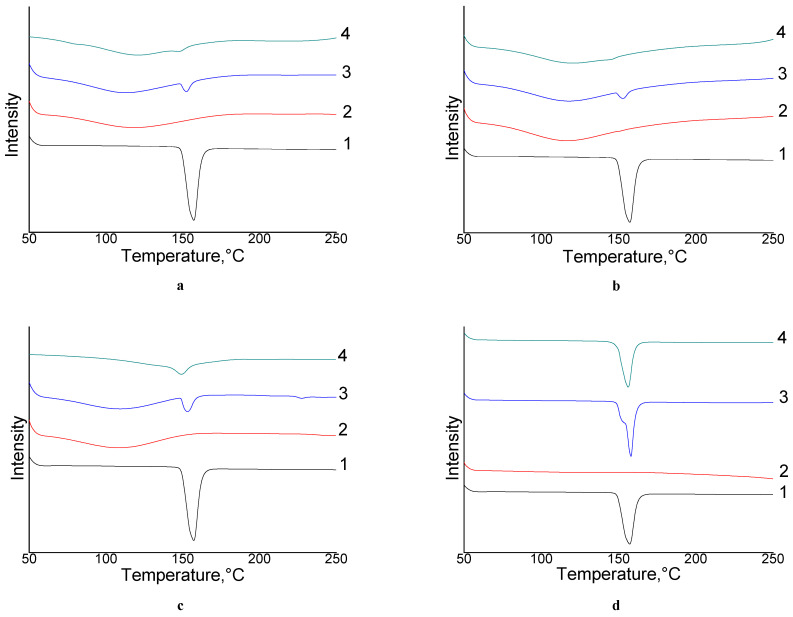
DSC thermograms of NIM API (**a**,1); AG (**a**,2); NIM/AG (1/10 mass relations) physical mixture (**a**,3) and solid dispersion of NIM/AG (1/10 mass relations) treated in, in roll mill for 24h-(**a**,4); NIM API-(**b**,1); Na_2_GA API-(**b**,2), NIM/Na_2_GA (1/10 mass relations), physical mixture-(**b**,3) and solid dispersion of NIM/Na_2_GA (1/10 mass relations) treated in roll mill for 24h-(**b**,4); NIM API-(**c**,1), HP-β-CD API-(**c**,2); NIN/HP-β-CD (1/1mol relation) physical mixture-(**c**,3) and solid dispersion of NIM/HP-β-CD (1/1mol relation) treated in roll mill for 24h-(**c**,4); NIM API-(**d**,1), MgCO_3_ API-(**d**,2); NIM/MgCO_3_ (5/1 mass relations) physical mixture-(**d**,3); and solid dispersion of NIM/MgCO_3_ (5/1 mass relations) treated in roll mill for 24h-(**d**,4).

The DSC thermograms of free NIM, mechanically treated NIM, physical mixtures (PMs), and NIM SD are shown in Figure 2a–d.

**Figure 3:**
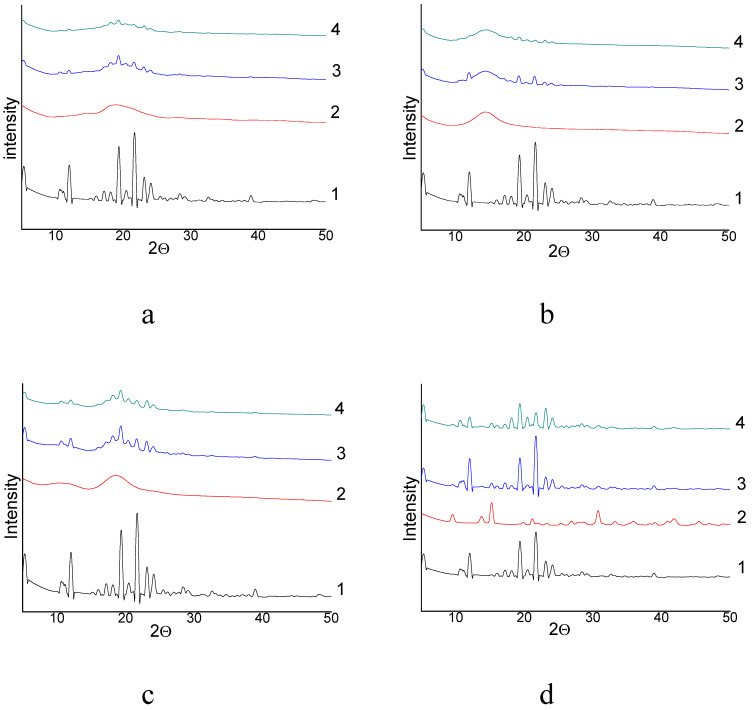
X-ray diffractograms of NIM API-(**a**,1); AG API-(**a**,2); NIM/AG (1/10 mass relations) physical mixture (**a**,3) and solid dispersion of NIM/AG (1/10 mass relations) treated in, in roll mill for 24 h-(**a**,4); NIM API-(**b**,1); Na_2_GA API-(**b**,2), NIM/Na_2_GA (1/10 mass relations), physical mixture-(**b**,3) and solid dispersion of NIM/Na_2_GA (1/10 mass relations) treated in roll mill for 24 h-(**b**,4); NIM API-(**c**,1), HP-β-CD API-(**c**,2); NIN/hp-β-cd (1/1mol relation) physical mixture-(**c**,3) and solid dispersion of NIM/HP-β-CD (1/1mol relation) treated in roll mill for 24 h-(**c**,4); NIM API-(**d**,1); MgCO_3_ API-(**d**,2); NIM/MgCO_3_ (5/1 mass relations) physical mixture-(**d**,3) and solid dispersion of NIM/MgCO_3_ (5/1 mass relations) treated in in roll mill for 24 h-(**d**,4).

The DSC curves of free NIM exhibited endothermic peaks around 143.5 °C with melting heat (MH) value of 109.9 J/g, which corresponded to its intrinsic melting points (MP) of NIM API and suggested its crystalline structure as far as relatively thing reflexes in RFA diffractograms (see Figure 3). The endothermic peak and RFA diffractograms of mechanically treated NIM without excipients are practically unchanged. However, the intensity of NIM peak was decreased significantly in NIM SD with milling time from 2 h to 24 h, as well as MH values decreased from initial melting heat to a minimal value that is difficult be calculated, indicating that NIM had possibly converted to a mainly amorphous state or probably partly dispersed in molecular form in the bulk phase of organic carrier during mechanochemical process. X-ray diffractograms of free NIM, milled NIM, excipients and NIM SD are shown in Figure 3a–d.

**Figure 4:**
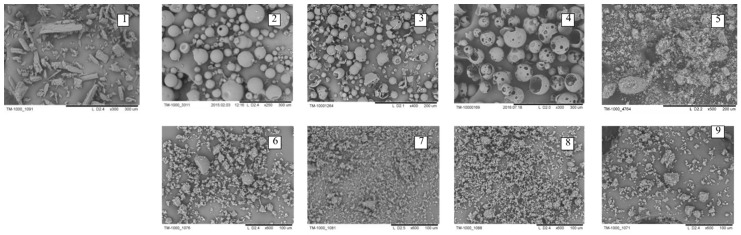
Electron micrographs of NIM API-(**1**); AG API-(**2**); Na_2_GA API-(**3**); HP-β-CD API-(**4**); MgCO_3_ API-(**5**); NIM/AG (1/10mass relations) solid dispersion, treated in roll mill for 24 h-(**6**); NIM/Na_2_GA (1/10 mass relation) solid dispersion, treated in roll mill for 24 h-(**7**); NIM/HP-β-CD (1/1mol relations) solid dispersion, treated in roll mill for 24 h-(**8**); NIM/MgCO_3_ (5/1, in roll mill for 1h) solid dispersion, treated in roll mill for 24 h-(**9**).

Free NIM showed several thing characteristic peaks at angles within 40°, indicating its crystalline form. However, the characteristic crystalline peaks of NIM were practically stable for pure mechanically treated NIM and significantly decreased in the diffractogram of NIM SD, and even practically disappeared in the 24 h milled NIM SD with Na_2_GA. The decreasing and lack of the characteristic crystalline peaks and the decrease or lack of endothermic peaks in the diffractogram and thermogram of NIM SD, respectively, were all consistent with the conversion of the formulation to a high energy partly or fully amorphous dispersion. One should to note, that in case of using AG and HP-β-CD adjuvants, the NIM substance stay partly crystalline in contrast of our previous investigations of similar mechanochemically obtained SD of curcumin, simvastatin and other API [22,39], probably because of more polar structure of NIM molecule. The electron micrographs of initial substances, milled NIM and obtained SDs are shown in Figure 4.

It could be clearly seen that the initial pure NIM and excipients had characteristic crystalline and intact shape. However, under mechanochemical milling processing, the destruction of the crystalline of NIM and ordered particles of MgCO_3_, HP-β-CD, AG, Na_2_GA occurred followed by the formation of polydisperse particles with irregular shape. As milling time was prolonged from 2 h to 24 h, the particle dispersed much more homogeneously, and aggregated which is charactering for solid dispersion’s formation Figure 4(1–9). In all mechanochemically obtained solid dispersions a NIM content varied between 98–100% from theoretically calculated, as has been shown by HPLC assay.

### 2.2. Characteristics of NIM SD in Solutions

#### 2.2.1. Intrinsic Solubility of NIM from Mechanically Treated Samples

The solubility of NIM was facilitated with varied extent in formulation of PM and mechanical treated compositions/complexes by comparison with unprocessed drugs (Table 1).

For mechanical treated solid dispersions/compositions, the phenomenon of solubilization is probably due to ionization of NIM, as far as due to the formation of inclusion complexes of NIM with HP-β-CD, AG and incorporation into micelles of GA which displays an internal hydrophobic cavity. In addition, the contents of drugs in complex were all nearly 100% from theoretically calculated. It indicated that during mechanical treatment, there was no significant destruction and loss of NIM in obtained SDs. For mechanically treated NIM API there was no practically change of solubility. In contrast, NIM milled with excipients showed increase of solubility in approximately 2.6-107 folds. The most increasing of NIM solubility (in 107 folds) was reached from SD with MgCO_3_, probably obliges to ionization and salt formation. NIM-HP-β-CD/AG/Na_2_GA systems, the mechanically processed SD exhibited not so high but sufficient increase of aqueous solubility (in 2.6–9.5 folds), too. Additionally, NIM-HP-β-CD/AG/Na_2_GA physical mixtures (PM) in water were weakly then from mechanochemically obtained SDs. One should note that, as a rule, mechanochemically synthesized complexes are more stable, than obtained from liquid state [20]. So, solid mechanochemical way has advantages comparably relative technology which uses liquids.

Unfortunately, we couldn’t directly compare our data with results published earlier for NIM/HP-β-CD complexes [9] because there was only a dissolution of tablets study and no measurements of intrinsic SDs solubility. Our thoughts are that investigations of intrinsic solubility are more correct for research articles because dissolution measurements should be connected with especial dosage forms and are necessary for applied studies.

#### 2.2.2. ^1^H-NMR Spectroscopy of the Aqueous Solutions of NIM and Its Solid Dispersions

In the present study the NMR relaxation technique was applied to prove the formation of NIM inclusion complexes after dissolution of SD in aqueous solutions. Earlier we have shown that the spin-spin nuclear relaxation time T_2_ of a drug molecules (carotenoids lutein and zeaxanthin [48], pesticides [49,50,51], anthelmintic drugs albendazole and praziquantel [10,12,44], curcumin [43,52], atorvastatin and simvastatin [42,53], and others [54]) is extremely sensitive to the mobility of the molecules in solution. Formation of inclusion complex with AG, GA or CDs results in a decrease of the diffusion mobility of molecules and leads to decrease in the observed relaxation time of corresponding protons. Thus, T_2_ data could be used as an indicator of whether a drug molecule exists in a free or bound state.

##### Self-Association of NIM in Aqueous Solutions

**Figure 5:**
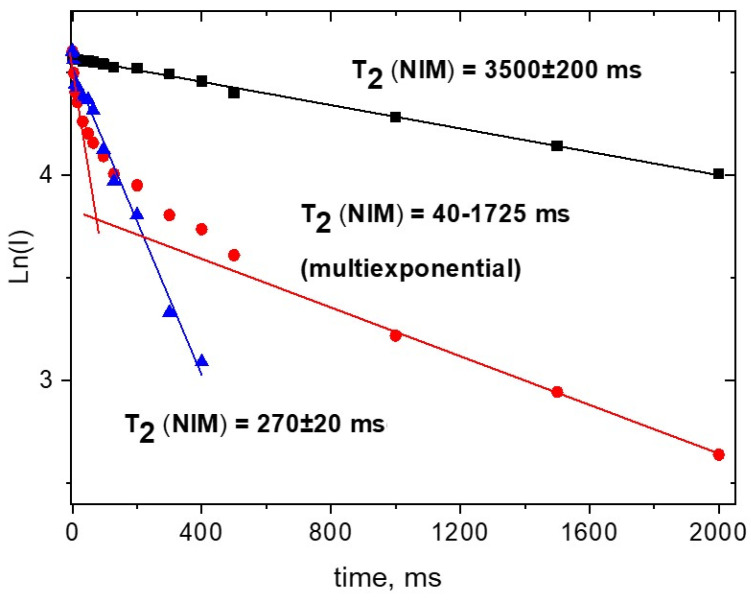
Kinetics of the echo signal decay of NIM protons (on a logarithmic scale, the 4-H and p-H protons give the same echo signal decays) at different methanol-water ratios at 30 °C. ▲—25% of MeOD; ●—50%; ■—100%.

In the present study it was found that the spin-spin relaxation time of NIM protons in aqueous solution of pure drug is much shorter (~0.3 s) than the characteristic time for free molecules (1-3 s) [8,33,55]. We assume that this effect is due to the self-association of NIM molecules in aqueous solutions. Figure 5 shows that the relaxation time of NIM protons decreases considerably with increase of water content in methanol solution.

Earlier, the same effect of drugs self-association was detected by NMR relaxation technique for praziquantel and curcumin aqueous solutions [12,52]. Actually, according to Figure 5, in 75% aqueous solutions a more than 10-fold shortening of T_2_ occurs. This value corresponds to a lowering of rotational diffusion mobility to nearly the same times. Taking in mind, that according Stocks-Einstein equation, D_rot_~1/d^3^ we can assume that the volume of NIM cluster (V~d^3^) should exceed the volume of single molecule at least in 10 folds. At 50% of water the multiexponential decay kinetics of NMR signals has been detected. It points to the coexistence of clusters of different sizes in such environment.

##### Interaction of Nimesulide with Na_2_GA in Aqueous Solutions

**Figure 6:**
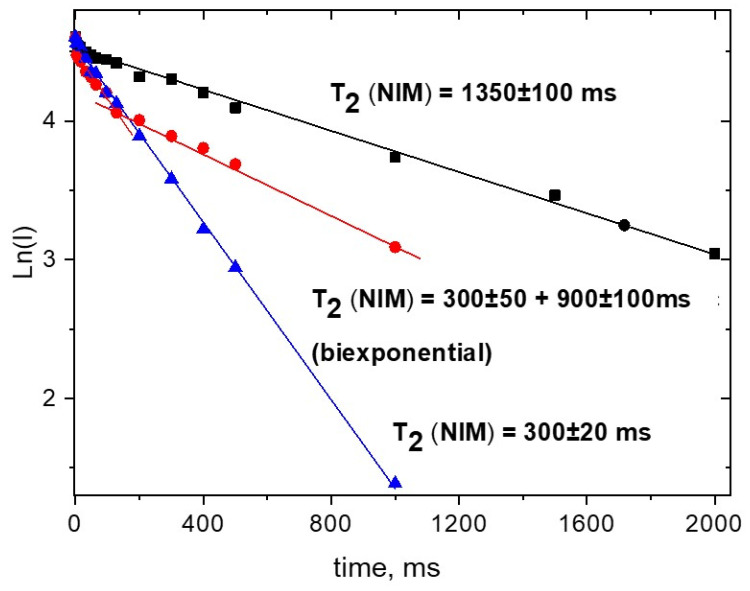
Kinetics of the echo signal decay of the protons of NIM (protons 4-H and p-H, give the same decays in the echo signal) in 1% solution of 1:10 SD with Na_2_GA at different ratios of methanol-water mixture at 30 °C. ▲—10% of MeOD; ●—25%; ■—50%.

The preparation of NIM solution from SD with Na_2_GA does not shows any clusters formation. The observed relaxation time is ~2 times lower than for free molecule, which is characteristic value for inclusion complex with GA dimers (Figure 6).

As it was described earlier [39,56,57,58,59], with increasing the water content in alcohol-water mixture, glycyrrhizic acid forms the micelles. In the present study, this was confirmed by the further decrease of diffusional mobility and relaxation time of NIM (up to 300 ms) due to inclusion of NIM into the micelles. Additionally, a formation of micelles was confirmed by gel permeation HPLC (see for example our previous article [12]).

##### Inclusion Complex Formation of NIM with HP-β-CD in Water Solution

**Figure 7:**
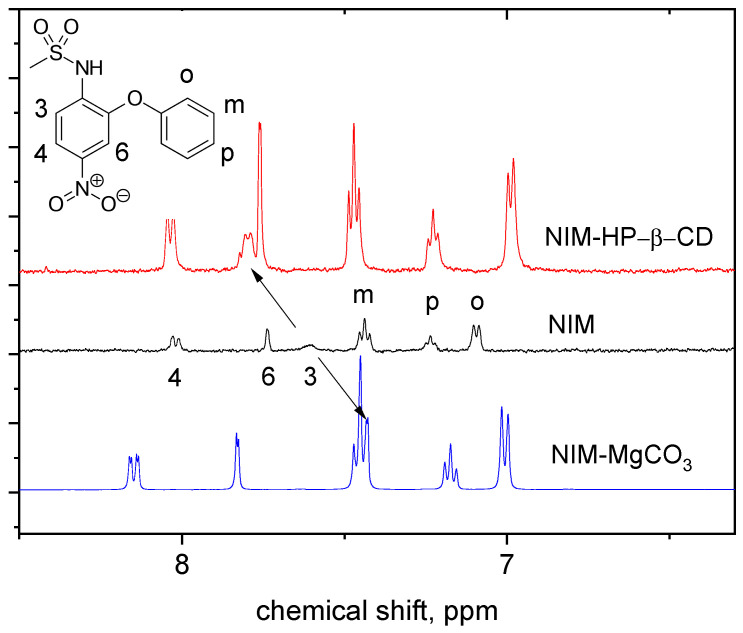
^1^H-NMR spectra of NIM in the absence and presence of HP-β-CD (20 mM) in 5% methanol, and a chelate complex with MgCO_3_ in water at 30 °C.

Most often, complexation with cyclodextrins is proved by a change in the chemical shifts of the protons of the guest molecule. For the complex of NIM with HP-β-CD, a change in the chemical shifts of protons on both cycles A and B was observed (Figure 7), which can mean the presence of two types of inclusion complex. The strongest shift is observed for 3-H protons, which can be explained by the desolvation of NIM during complexation. We note that this effect is observed for all complexes of NIM: with HP-β-CD, with AG, and with Na_2_GA. From the concentration dependence of NIM chemical shift one can obtain the information about the stability constant and stoichiometry of the inclusion complex. In this study, the stability constant and stoichiometry of the complex were calculated using Benesi-Hildebrant diagram (Equation (1)):

Δ*δ_max_*/Δ*δ* − 1 = 1/*[CD]*^n^ × 1/*K* (1)

where *K* is the stability constant of the complex, and Δ*δ* is change of chemical shift.

Plotting the concentration dependence in the coordinates Δ*δmax*/Δ*δ* versus 1/*[CD]* gives a linear dependence for fragments A and B of NIM. It confirms the assumption about the possibility of the formation of two types of complex with stoichiometry 1:1 (n = 1 in Formula (1)). The stability constants for cycles A and B at 30 °C are 140 and 490 M^−1^. Measurement of K values at different temperatures at 30–50 °C allows us to calculate the thermodynamic parameters of the HP-β-CD complex. From the dependence of R × lnK vs. 1/T using formulas (Equation (2)) and (Equation (3)), one can calculate the main thermodynamic parameters of complexation: the change in the Gibbs energy, enthalpy, and entropy:

*R* × *lnK* = −Δ*H*^0^/*T* + Δ*S*^0^ (2)

Δ*G*^0^ = Δ*H*^0^ − *T*Δ*S*^0^ (3)

The obtained values of Δ*G*^0^, Δ*H*^0^, and Δ*S*^0^ at 303 K are equal, respectively, for cycle A: Δ*G*^0^ = −3.7 kcal/mol, Δ*H*^0^ = −10.5 ± 0.5 kcal/mol, and *T*ΔS^0^ = −6.7 ± 0.3 kcal/mol; and for cycle B: Δ*G*^0^ = −3.0 kcal/mol, Δ*H*^0^ = −12.2 ± 1.0 kcal/mol, and *T*Δ*S*^0^ = −8.2 ± 0.5 kcal/mol. Thus, the enthalpy factor plays a decisive role in the stability of the complex. However, the low values of the stability constants of the complexes are caused by the compensation of the enthalpy factor by the loss of entropy.

##### Inclusion Complex Formation of NIM from SD with Polysaccharide Arabinogalactan in Water Solution

As already mentioned, for all complexes, including AG, a change in the chemical shifts of the protons of NIM in the complex was observed. Additionally, evidence of complexation was obtained by NMR relaxation method. The measured relaxation times of the aromatic protons 4-H and m-H in water are 125 ms and 130 ms, which are close in value to the relaxation times of protons of other drug molecules in complexes with AG (ibuprofen, valsartan, etc., 100-140 ms) [35,36]. Due to its low solubility, uncomplexed NIM does not contribute to the observed NMR signal of the complex in water. As it was mentioned above, free NIM molecules have significantly longer relaxation times (T_2_ = 3500 ms).

##### Chelate Complex Formation of NIM with MgCO_3_ in Water Solution

The mechanochemically prepared composition of NIM with MgCO_3_ (5:1) showed the best solubility in water among all the studied formulations. In this case, in the NMR spectrum of NIM, a significant change in the chemical shift of 3-H protons is observed, which indicates the formation of a chelate complex with a magnesium ion. A distinctive feature of the chelate complex is the shift of the 3-H proton in the opposite direction compared to the inclusion complexes (Figure. 7). This is due to the influence of the positive metal ion on the electron density distribution in the aromatic ring [60]. Similar changes are observed in the NIM/CaCO_3_ chelate complex. Note that the literature contains examples of chelate complexes of nimesulide with silver, copper and other metals [61,62]. The calculations carried out in these works showed that the chelating group in the NIM molecule is the NH-SO_2_ fragment. In this case, the relaxation times of NIM protons measured in water are approximately coincide with those for the free molecule of NIM in methanol: (T_2_ = 3000 ms and 3370 ms for 4-H and p-H vs. 3500 ms), which indicates the absence of self-associates in the solution of chelate complex.

The formation of a chelate complex is also accompanied by a change in the absorption spectrum of the NIM solution. To calculate the stoichiometry and stability constant of the chelate complex, the concentration dependence of the change in the optical density of an alcohol solution of NIM on the concentration of magnesium ions was measured. Simulation of the experimental data using a previously developed approach (see Experimental Section 2.1 for the calculation details) allowed us to conclude that a 1:1 stoichiometry complex with a stability constant of 22,300 ± 7400 M^−1^ was formed.

We can summarize that the NMR relaxation technique performed in present study have allowed to prove the formation of NIM inclusion complexes with HP-β-CD, polysaccharide arabinogalactan and saponin glycyrrhizin after dissolution in water of mechanochemically prepared solid compositions. In the case of composition with MgCO_3_, the water soluble chelate complex of NIM with Mg ion was formed.

#### 2.2.3. In Vitro Permeation Study

**Figure 8:**
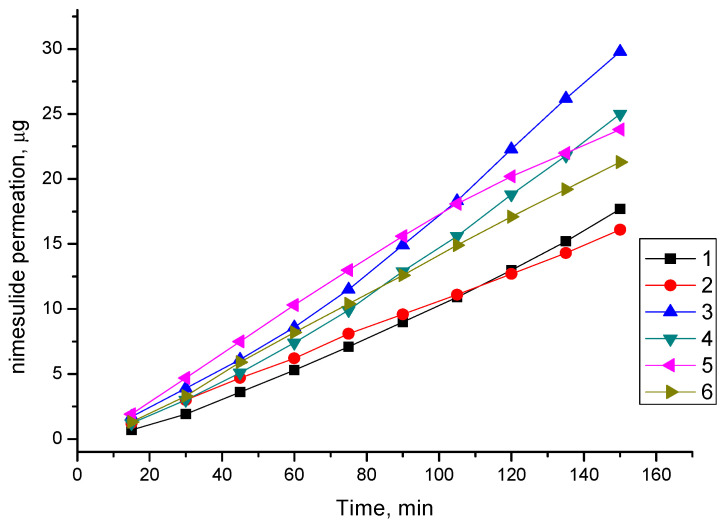
Permeation profile of nimesulide (1) and complexes: NIM/MgCO_3_(5/1) (2), NIM/AG (1/10) (3), NIM/Na_2_GA (1/10) (4), NIM/HP-β-CD (1/1mol) (5), NIM/HP-β-CD (1/2 mol) (6).

A PAMPA assay enabled fast determination of the trends in the ability of the compounds to permeate membrane by passive diffusion and thus was suitable for screening potential drugs [63,64,65]. In the plots (Figure 8), it can be seen that the amount of NIM from mechanochemically treated complexes permeated is higher than pure NIM, as far as from SD with MgCO_3_ indicating that the co-grinding complexes with HP-β-CD, AG and Na_2_GA have enhanced the permeation/flux/mass transport of NIM across an artificial membrane compared to that of the pure drug.

Drug molecules encounter two types of resistance when they permeate through artificial membranes, i.e., membrane resistance (RM) in the lipophilic membrane and diffusion resistance in the unstirred water layers (UWL) adjacent to surfaces of the lipophilic membrane [66,67]. Earlier it was found that hydrophilic cyclodextrins could exert an improvement in drug permeation, which was associated with the ability of HP-*β*-CD to transport a drug molecule though the UWL so that it was brought in close proximity to the lipophilic membrane.

The NIM low permeation throw artificial membrane from SD with MgCO_3_ seen slightly surprising, because it had highest solubility, which is more than 10 folds higher than from another SDs. The only reasonable explanation is that in this case increasing of solubility reaches by ionization and salt formation and more hydrophilic and charged forms of NIM molecules cannot be accepted by hydrophobic membrane.

### 2.3. In Vivo Tests

#### 2.3.1. Anti-Inflammatory Study of NIM and Its Compositions

**Figure 9:**
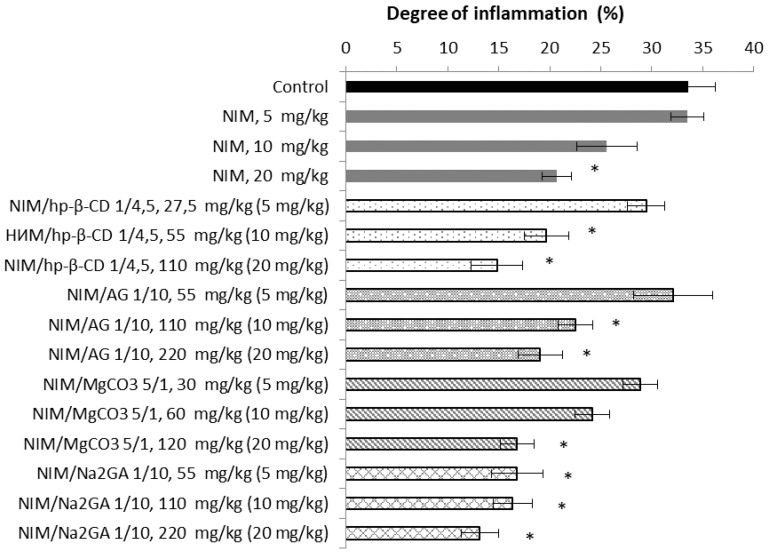
Anti-inflammatory effect of NIM compositions with AG, Na_2_GA, HP-β-CD and MgCO_3_ in the “histamine edema” test. Doses in brackets are NIM’s doses in respective complex. * *p* < 0.05 compare to control.

Obtained NIM compositions were tested for anti-inflammatory activity in a histamine edema model. The dose of NIM in all cases was 5, 10, and 20 mg/kg after oral single administration. In the case of NIM and the complex with MgCO_3_, a significant effect was found only at the highest dose of 20 mg/kg, while for all others the dose of 10 mg/kg also became effective. This suggests that AG, Na_2_GA and HP-β-CD contribute to an increase in bioavailability not only by increasing solubility in water, as in the case of MgCO_3_, but also by interacting with the membrane of epithelial cells of the gastrointestinal tract [11,45,68]. Pure NIM was mixed with Tween 80 before dissolving in water, which increases solubility due to the formation of micelles [69] and in the case of compositions, including those with MgCO_3_, only water was used. The greatest decrease in the effective dose was achieved with the introduction of the complex with Na_2_GA, in this case, a significant anti-inflammatory effect manifested itself in the entire range of doses used (Figure 9).

#### 2.3.2. Pharmacokinetics of NIM and Its Compositions

**Figure 10:**
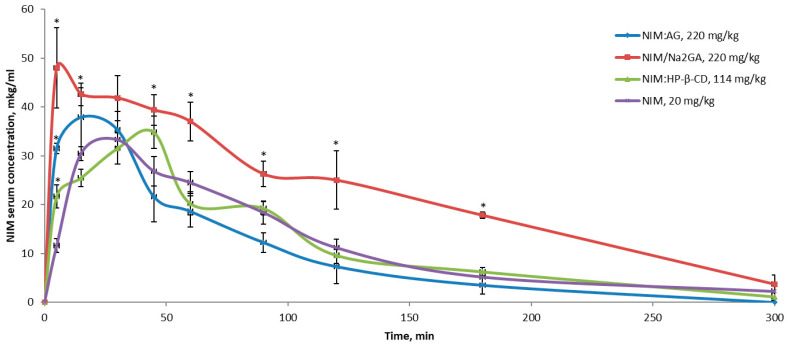
Nimesulide serum concentration after a single *per os* administration of its compositions with AG, Na_2_GA and HP-β-CD. NIM dose for all substances 20 mg/kg. * *p* < 0.05 compare to NIM, 20 mg/kg.

For the study of pharmacokinetics, only complexes with AG, Na_2_GA, HP-β-CD were taken at a dose of 20 mg/kg in term of NIM, as they showed the best anti-inflammatory effect. NIM complex with MgCO_3_ was excluded from this study since it didn’t show the increase in the anti-inflammatory effect. A single administration of the complexes noticeably increases the concentration of NIM in the blood plasma already 5 min after administration (Figure 10). The concentration at this point increases in the following order NIM > NIM:HP-β-CD > NIM:AG > NIM:Na_2_GA, so this increase in the NIM’s bioavailability can be also extrapolated on the lower dose of the NIM complexes 10 mg/kg (in terms of NIM) and explain their better anti-inflammatory effect in comparison with pure NIM in this dose (Figure 9). Besides, it should be emphasized that in all cases of the pure NIM introduction the Tween 80 as the solubilizer was used which means that without any auxiliary components it would not show any pharmacological effect. Pharmacokinetic profile of NIM:AG is generally similar to pure NIM, except for faster absorption, while their AUC is the same. NIM from the complex with HP-β-CD is absorbed with some delay, however, its pharmacokinetic parameters are similar to pure NIM and complexes of NIM with AG (Table 2). The greatest differences are observed after the introduction of NIM:Na_2_GA. In addition to the fastest absorption, Na_2_GA provides a significant increase in C_max_ and the bioavailability (AUC) of NIM after a single introduction (Table 2). This feature of pharmacokinetics is responsible for the best anti-inflammatory effect of this complex, observed in the histamine edema test for all doses used.

Thus, of all the tested carriers, Na_2_GA showed the greatest efficiency in increasing the bioavailability and decreasing the effective NIM anti-inflammatory doses.

## 3. Materials and Methods

### 3.1. Materials

NIM (Well Green Technology Co. Ltd., Xi’an, China) of pharmaceutical grade was used without further purification. AG from Siberian Larch wood (purity of AG > 99.5%, moisture—0.5%, the content of phenolic impurities—0.15%, MW—14,300 Da) was provided by the company Wood Chemistry (Irkutsk, Russia). Na_2_GA (CFS, 98%) was purchased from Shaanxi Sciphar Biotechnology Co. Ltd. (Xi’an, China). All other chemicals like HP-β-CD and MgCO_3_ were of pharmaceutical grade and used without further purification.

### 3.2. Preparation of NIM Composition with Excipients

Mechanical treatment of NIM compositions with HP-β-CD, AG, Na_2_GA and MgCO_3_ was carried out in a VM-1 roll mill with a cylindrical vessel which was coated with Teflon and possessed 300 mL volume. Acceleration of grinding bodies is 1 g (free fall). Rotational speed of cylindrical vessel is 157 rpm. Steel balls (diameter 22 mm, 675 g load) were used as grinding bodies. The total load of the treated powders mixture was 22 g. The duration of mechanical processing was from 2 to 24 h—(2, 4, 8, 16, and 24 h). To prepare the solid dispersions, we used NIM/HP-β-CD molar ratio 1/1 and 1/2, NIM/AG mass ratio 1/10, NIM/Na_2_GA mass ratios 1/10 and NIM/MgCO_3_ mass ratio 5/1.

Physical mixtures were prepared by shaking (for about 1 h) the previously noted powdered compounds in closed test tube.

### 3.3. HPLC Analyses

An Agilent 1200 HPLC system (Agilent Technologies, Palo Alto, CA, USA) was used to determine the concentration of NIM. The HPLC system was equipped with two pumps and a diode-array detector. Chromatographic analysis was performed on a reverse phase column (5 μm, 4.6 × 50 mm, Zorbax Eclipse XDB-C18) at 30 °C. The mobile phase consisted of phosphate buffer (pH 7.0) and acetonitrile (55:45, *v/v*) and the detector operated at 400 nm. The flow rate was 1 mL/min and the injection volume for each sample was 5 μL.

### 3.4. Content Test for NIM and Its Compositions

To determine the content of NIM in the obtained compositions, weighed samples (10 mg) were dissolved in 25 mL of ethanol. In all cases, all components of complexes were completely dissolved. The samples were then suitably diluted and assayed by HPLC.

### 3.5. Intrinsic Solubility Study for NIM and Its Complexes

To determine the water solubility of NIM, the mechanical treated products, which were withdrawn after different milling times, were separately put into a 50 mL flat-bottomed flask with 10 mL of distilled water. Then the flasks containing the mixtures were kept in a shaking incubator for 1, 2, 3, 6, 16 h with 200 rpm at +37 °C. It was shown that constant concentration of NIM in solution was reached at time of dissolution less than 3 h and this doesn’t change till at least 16 h. The noted time (3 h) was used in further experiments. The resulting suspensions were placed in centrifuge tubes and then centrifuged at 12,000 rpm (14,800 *g*) for 5 min. The samples were then filtered using a paper filter and analyzed by the abovementioned HPLC method.

### 3.6. Powder X-ray Diffraction (XRD)

X-ray diffraction analysis (XRD) of solid powdered complexes was carried out on a DRON-4 instrument (Burevestnik, St. Petersburg, Russia) using CuKα radiation, counter speed 2 deg/min, range of intensity measurement-1000.

### 3.7. Differential Scanning Calorimetry (DSC)

Thermal analysis of the samples was carried out by differential scanning calorimetry (DSC) on a DSC-550 device (Instrument Scientific Specialists Inc., Omaha, NE, USA) in an Ar atmosphere, with the temperature regimen +20–+250 °C and the heating rate 10 °C/min.

### 3.8. Scanning Electron Microscopy (SEM)

Electronic images were acquired using a TM-1000 microscope (Hitachi, Tokyo, Japan). Coating of samples with gold was performed using a JFC-1600 auto fine coater (JEOL, Tokyo, Japan). The coating parameters were as follows: sputtering time 30 s, amperage 30 mA, and film thickness 15 nm.

### 3.9. Gel Permeation Chromatography (GPC)

The molecular weight distribution of the AG and micelles of Na_2_GA was analyzed by gel permeation chromatography on the Agilent 1200 chromatograph with PL aquagel-OH 40 column at +30 °C with a refract- metric detector. The solvent were a 0.1 M aqueous solution of LiNO_3_ or NaN_3_, and the flow rate was 1 mL/min. The calibration was based on standard dextran with molecular weights of 1, 5, 12, 25, 80, 150, 270, and 410 kDa. The Agilent GPC Date Analysis software was used to process the results.

### 3.10. ^1^H-NMR Spectroscopy

^1^H-NMR spectra were recorded on an Avance III 500 MHz spectrometer (Bruker, Rheinstetten, Germany) in D_2_O and CD_3_OD (99.8%, Sigma-Aldrich, Moscow, Russia) solutions as well as in their mixture. Spin-spin relaxation time T_2_ was measured by the Carr-Purcell-Meiboom-Gill (CPMG) pulse sequence.

### 3.11. In Vitro Parallel Artificial Membrane Permeability Assay (PAMPA)

PAMPA experiments were carried out in 12-well filter plates (polycarbonate membrane, 12 mm diameter inserts, 0.4 μm pore size, 1.12 cm^2^ area, Corning Inc., Corning, NY, USA). The ability of compounds to diffuse from a donor compartment into an acceptor compartment is evaluated. The artificial membrane was first impregnated by carefully pipetting 60 μL of the 5% (*v/v*) hexadecane in hexane solution to each of the wells of the donor plate. The wells were then placed into a fume hood for 1 h to ensure complete evaporation of the hexane. After the hexane had evaporated, 1.5 mL of water was added to each of the wells of the acceptor plate. The hexadecane treated donor plate was then placed on top of the 12-well acceptor plate. Then, 0.5 mL of NIM or its complexes in water were added to each well of the donor plate, and the resulting PAMPA device was incubated at room temperature (25 °C) and shaken for 6 h with 200 rpm. Samples (1 mL) were collected from the acceptor plate at appropriate time points (0.5, 1.0, 1.5, 2, 3, 4, 5 and 6 h) and were analyzed.

### 3.12. Optical Absorption Studies the NIM-Mg^2+^ Chelate Complex

Optical spectra of NIM API solution and its chelate complexes with Mg^2+^ ions were measured in using SF-2000 (Spectrum, Sant-Peterburg, Russia) spectrophotometer in 1 cm quartz cuvette using wavelength 400 nm. The calculation of the parameters of chelate complexes was carried out on the basis of a complexation model that takes into account the possibility of sequential formation of complexes of different stoichiometry (Equations (4) and (5)):

L + M ↔ LM

L + LM ↔ L_2_M

L—the light absorbing ligand, M­metal.

Stability constants for 1:1 and 2:1 complexes are:

(4) $$K_{1}=\frac{\left[LM\right]}{\left[L][M\right]}$$

(5) $$K_{2}=\frac{\left[L_{2}M\right]}{\left[LM][L\right]}$$

For calculation details see [70]. Using this approach, the extinction coefficients of 1:1 complex and 2:1 complex, as well as K_1_ and K_2_ can be calculated by least squares method and method covariance matrix.

### 3.13. In Vivo Study

#### 3.13.1. Animals

CD-1 mice weighing 20–25 g were obtained from the vivarium of the Institute of Cytology and Genetic SB RAS. Animals were kept under standard conditions with free access to water and food with humidity and temperature-controlled rooms with light and dark 12 h cycles. All manipulations with animals was carried out in strict accordance with the legislation of the Russian Federation, Order of the Ministry of Health of the Russian Federation No. 199n of 04/01/2016 and the provisions of Directive 2010/63/EU of the European Parliament and of the Council of the European Union of 09.22.2010 on the protection of animals used for scientific purposes.

#### 3.13.2. Pharmacokinetic Study

Mice were randomized by weight and divided into groups and deprived of food for 12 h prior to the experiment. Free NIM, NIM/Na_2_GA (1/10 mass relations), NIM/AG (1/10 mass relations), NIM/HP-β-CD complexes (1:1 mole relations), at a dose equivalent to 20 mg/kg NIM were introduced by oral gavage. Complexes were dissolved in distilled water. Free NIM was mixed with 2 drops of Tween 80 and then dissolved in distilled water. Blood samples were withdrawn after decapitation at 5, 15, 30, 45, 60, 90 120, 180 and 300 min (*n* = 5 for each time point) after introduction. Then, all the blood samples were centrifuged for 15 min at 1640× *g* and the supernatant serum fraction was transferred to empty tubes and kept frozen at −20 °C prior to analysis. The preparation of a blood sample was carried out according to the method described by [71]. Briefly, the mouse plasma sample (0.2 mL) was transferred into glass test tube. 3 mL mixture solvent of ethyl acetate and hexane (90:10%, *v/v*) was added to the tubes and was vortexed for 3 min. Following a centrifugation for 7 min, 1.5 mL of organic phase was removed to another tube and evaporated. The dry residue was reconstituted with 100 μL of acetonitrile, vortexed for 30 s and 20 μL of the mixture was injected into the chromatograph. However, in contrast with noted reference we use HPLC conditions as described in HPLC analyses of this section. Standard pharmacokinetic parameters for the NIM such as maximum concentration (C_max_), half-life (T_1/2),_ T_max_, area under the concentration-time curve from time zero to infinity (AUC_0–inf_obs_), were calculated by linear trapezoidal method using the PKSolver, a freely available menu-driven add-in program for Microsoft Excel suggested by [72].

#### 3.13.3. Anti-Inflammatory Study

“Histamine swelling” was reproduced by introducing of 0.05 mL of 0.1% histamine solution under aponeurosis of the hind paw. The test compounds were administered by oral gavage 1 h before administration of inflammatory agent. The swelling degree was evaluated after 5 h by comparison the legs mass with inflammatory agent (m_1_) and without it (m_2_). The degree of inflammation (ID) for each mouse was calculated by the following formula (Equation (6)):

(6) $$ID=\frac{m_{1}-m_{2}}{m_{2}}\times 100\%$$

Nimesulide was introduced in three doses–5, 10, 20 mg/kg, all tested complexes were introduced in the same doses in terms of NIM.

#### 3.13.4. Statistical Analysis

The statistical analysis was performed using Mann–Whitney U test. Data shown as Mean ± SEM. *p* < 0.05 was considered to be statistically significant.

## 4. Conclusions

In this study we investigated for the first time the possibility of improving the solubility and the bioavailability of NIM by preparing solid dispersions with HP-β-CD, polysaccharide arabinogalactan, disodium salt of glycyrrhizic acid and MgCO_3_ using one-step solid state mechanochemical technology. It was shown that milling of pure NIM API without additives didn’t sufficiently help improve its aqueous solubility. The physical properties of NIM SD in solid state were characterized by differential scanning calorimetry, X-ray diffraction and SEM studies. The characteristics of the water solutions, which are formed from the obtained solid dispersions, were analyzed by HPLC for intrinsic solubility and ^1^H-NMR spectroscopy. It was shown that solubility increases for all compositions under investigation. These phenomena are caused by inclusion complexation with organic auxiliary substances—HP-β-CD, AG, Na_2_GA, which was shown by ^1^H-NMR relaxation methods. In the case of MgCO_3_, ionization and salt formation were proposed. The PAMPA assay was used for predicting passive intestinal absorption. It was found that mechanochemically obtained complexes with HP-β-CD, AG, Na_2_GA show enhanced permeation of NIM across an artificial membrane compared to that of the pure NIM and NIM/MgCO_3_ SD. Our view is that charged NIM molecules as far as its chelate complexes with Mg^2+^ ions have higher hydrophilicity and good solubility in water solutions which prevents their penetration throw lipophilic membranes.

The compositions were examined for anti-inflammatory activity on a model of histamine edema. It was found that complexes with HP-β-CD and AG can achieve anti-inflammatory effect in dose twice lower than NIM dose. In the case of NIM:Na_2_GA composition the effective dose can be reduced up to four times. This effect is due to the improved bioavailability of the NIM incorporated into the complexes with HP-β-CD, AG and most pronounced in case of Na_2_GA. MgCO_3_ just improve NIM’s water solubility but actually do not affect its pharmacological action.

Thus, the compositions of NIM with HP-β-CD, polysaccharide AG and disodium salt of glycyrrhizic acid obtained using the mechanochemical manufacturing method are a promising basis for the development of NIM-based preparations for oral administration, with reduced dose and high pharmacological effect.

## Figures and Tables

**Table 1:** Solubility characteristics of NIM SD and PM with different excipients.

Sample Content (Weights or Mole to Mole Ratios) and Time of Mechanical Treatment in Hours	Concentration of NIM in Water Solution, g/L	Increasing of Solubility, Times	pH of Solution
Nimesulid API	0.019	-	6.4
Nimesulide/AG, SD, 1/10, mass ratio, 4 h	0.049	2.6	6.7
Nimesulide/AG, PM, 1/10, mass ratio	0.19	-	6.7
Nimesulide/MgCO_3_, SD, 5/1, mass ratio, 1h	2.041	107	9.2
Nimesulide/MgCO_3_, PM, 5/1, mass ratio	1.24	65	9.3
Nimesulide HP-β-CD, SD, 1/1 mol ratio, 8 h	0.059	3.1	6.6
Nimesulide HP-β-CD, PM, 1/1 mol ratio	0.029	1.5	6.6
Nimesulide HP-β-CD, SD, 1/2 mol ratio, 4 h	0.065	3.4	6.7
Nimesulide HP-β-CD, PM, 1/2 mol ratio	0.045	2.4	6.7
Nimesulid/Na_2_GA, SD, 1/10, mass relation, 16 h	0.181	9.5	5.8
Nimesulid/Na2GA, PM, 1/10, mass relation	0.029	1.5	5.8

SD—mechanochemically obtained solid dispersion, PM—“physical mixture”.

**Table 2:** Pharmacokinetic parameters after a single *per os* administration of NIM compositions with AG, Na_2_GA and HP-β-CD. NIM dose for all substances 20 mg/kg. * *p* < 0.05 compare to NIM.

	NIM:AG	NIM:Na_2_GA	NIM:HP-β-CD	NIM
T_1/2_, min	99.94 ± 43.03	83.45 ± 17.99	74.59 ± 12.45	89.09 ± 20.81
T_max_, min	30.00± 10.66	32.50 ± 7.37	36.00 ± 3.67	34.29 ± 6.31
C_max_, mkg/mL	38.50 ± 2.50	49.39 ± 2.20 *	37.16 ± 3.41	38.43 ± 3.25
AUC _0-inf_obs_, mkg/mL*min	4033.30 ± 597.85	7534.44 ± 576.98 *	3898.39 ± 201.28	3978.88 ± 242.62

## Data Availability

Data is contained within the article.

## Sample Availability

Samples of the compounds are not available from the authors.
